# The Investigation of Laparoscopic Instrument Movement Control and Learning Effect

**DOI:** 10.1155/2013/349825

**Published:** 2013-07-24

**Authors:** Chiuhsiang Joe Lin, Hung-Jen Chen

**Affiliations:** Department of Industrial Management, National Taiwan University of Science and Technology, No. 43, Section 4, Keelung Road, Da'an District, Taipei 106, Taiwan

## Abstract

Laparoscopic surgery avoids large incisions for intra-abdominal operations as required in conventional open surgery. Whereas the patient benefits from laparoscopic techniques, the surgeon encounters new difficulties that were not present during open surgery procedures. However, limited literature has been published in the essential movement characteristics such as magnification, amplitude, and angle. For this reason, the present study aims to investigate the essential movement characteristics of instrument manipulation via Fitts' task and to develop an instrument movement time predicting model. Ten right-handed subjects made discrete Fitts' pointing tasks using a laparoscopic trainer. The experimental results showed that there were significant differences between the three factors in movement time and in throughput. However, no significant differences were observed in the improvement rate for movement time and throughput between these three factors. As expected, the movement time was rather variable and affected markedly by direction to target. The conventional Fitts' law model was extended by incorporating a directional parameter into the model. The extended model was shown to better fit the data than the conventional model. These findings pointed to a design direction for the laparoscopic surgery training program, and the predictive model can be used to establish standards in the training procedure.

## 1. Introduction

Laparoscopic surgery, or minimally invasive surgery (MIS), is performed increasingly and is the procedure of choice for a growing number of treatments in recent years [1–3]. In laparoscopic surgery, a surgeon performs a surgical operation by using instruments through three or more trocars (ports) into the abdominal cavity (each hole is about 10 mm in diameter) which permit the introduction of a camera-monitored telescope and two or more fine instruments to perform the operation in a similar manner as formerly performed in open surgery. Due to the small incisions, laparoscopic surgery has brought many benefits to patients. The reduction of pain, the shorter recovery time and hospital stay, and the earlier restitution of normal physiological markers have been proven objectively in many well-designed clinical studies [4–8]. This new approach requires, in comparison to open surgery, an additional spectrum of devices and technical support (lights sources, camera, control unit, insulator, video screens, etc.). Thus, laparoscopic surgery is highly advantageous for the patient. However, it is necessary for the surgeon performing such surgery to possess a high surgical skill.

Whereas the patient benefits from laparoscopic techniques, the surgeon encounters new difficulties that were not present during open surgery procedures [9–11]. These difficulties include impairments in depth perception, in the ability to develop mental models of the anatomical environments, and in perceptual-motor coordination. They also experience greater fatigue [12].

The view of the operative situation is displayed on a monitor that is widely separated from the field of action [13], so the surgeon has to overcome the natural instinct to direct the eyes to the activity of the hands. The two-dimensional viewing of a three-dimensional field has to be interpreted and synchronized to instrument movement [5, 14, 15]. This loss of binocular information leads to problems in hand-eye coordination and in cognitive mapping [16].

Compared with open surgery, depth perception is degraded in laparoscopic surgery because of several characteristics of the imaging technology. First, the camera image is two-dimensional and lacks the depth cue of binocular disparity [11]. Disparity is essential for judgments about relative depth [17] and for performance at near distances [18]. Second, the camera image provides a field of view (FOV) that is substantially smaller than the full FOV afforded by open surgery [11]. In the context of laparoscopic surgery, surgeons reported limited FOV as a factor that contributed to constraints and difficulties [19]. Third, the movement of the camera is limited because it is located in the patient's abdominal wall [20]. An assistant aims to keep the camera stationary to prevent the surgeon from experiencing spatial disorientation, fatigue, and nausea [21, 22].

When depth information is impoverished, as it is in laparoscopic surgery, surgeons putatively must “fill in” the missing information by developing mental models of the three-dimensional space from the images [23]. They also must perform mental operations on these mental models (e.g., mental rotations) that can contribute to response delays, errors, and cognitive workload [24]. In short, laparoscopic surgery requires different visuospatial skills and potentially greater cognitive processing demands than open surgery [25].

To overcome this lack of depth perception the operating surgeon uses a variety of monocular or two-dimensional cues, namely, light and shade, relative size of objects, object interposition, texture gradient, aerial perspective, and, most important, motion parallax [26]. These cues compensate somewhat for the lack of depth perception of two-dimensional vision but do not make up completely for the accuracy of the three-dimensional imaging. The surgeon often has to find the position of instruments by touching the organ or tissue to be cut or manipulated and so determine their position before using them. As a result, surgical tasks that take seconds during open surgery can take minutes during laparoscopic surgery [27].

The greatest ergonomic problem is the perceived inversion of movement from the handles to the working end of the surgical instrument. This perceived inversion of movement is caused by the “fulcrum effect” of the abdominal wall [28, 29]; for example, an external movement to the right by the surgeon's hand is displayed as a movement to the left on the monitor. This inversion affects both horizontal and vertical movements and is the normal laparoscopic condition under which all laparoscopic surgery operations are conducted. Thus, laparoscopic surgery creates discordance between the visual and proprioceptive systems. This causes incorrect sequencing of psychomotor output that requires a significant period of compensatory change [29].

In addition to those new difficulties encountered by surgeons during laparoscopic procedure, magnification of visual scale is another important optical property for laparoscopic surgery. When the user has adapted to the scale difference between the physical workspace and the display, magnifying the view scale of an operation would enhance fine movement control. Langolf et al. [30] found that it took less time to make very small-scale pointing movements when the hand-held pointer and target were viewed through a microscope. In human computer interaction field, Guiard et al. [31] also found users could acquire very small targets in a computer interface (i.e., <1 mm) with the aid of a zoom feature.

In a recent study, Bohan et al. [32] manipulated the visual scale of a pointing task with a mouse on the display while holding the physical movement scale constant. This condition simulates making fine control movements of a handheld tool while indirectly observing a magnified view of the objects and actions. Based on their experiment results, movement time decreased with increasing display scale. Unfortunately, this experimental configuration in which a mouse was used as a handheld tool to perform a two-dimensional target acquisition task was quite different from the manipulation of laparoscopic surgery. The effects of magnification on the three-dimensional human movement performance under fulcrum effect and indirect vision conditions were still unclear. 

 When the user has adapted to the scale difference between the physical workspace and the display, the larger visual scale of the task may augment movement control performance. However, the limited space and high magnification involved in minimally invasive surgery can also cause surgeons to lose sight of an instrument. This can occur when performing tasks such as tying a knot that requires the instruments to be pulled in opposite directions. The surgeon also loses sight of the instruments when they are exchanged through the body [33].

In sum, the performance of movements during tool use may vary with the visual magnification of the task on screen or the motion scaling of the effector on screen. Previous studies have indicated that the magnification levels were important in laparoscopic surgery. However, there remain discrepant results that need to be reconciled to provide a more complete understanding of performance when observing manual operations indirectly via a scaled-up view of the workspace. The first purpose of this research was to seek a better understanding the effects of visual magnification on human performance and control in operating a tool via indirect vision. We expected that the movement time and the throughput for pointing tasks with the fulcrum effect and indirect vision would be affected markedly by visual magnification levels.

As mentioned previously, stern conditions in performing a laparoscopic procedure result in new challenges to surgeons. Repeated training and practice can improve surgeons' skill to shorten duration of the laparoscopic procedures and prevent errors [34]. With respect to the fulcrum effect, Gallagher et al. [28] have demonstrated that for inexperienced individuals, simple laparoscopic cutting task performed under normal laparoscopic surgery viewing conditions resulted in a significantly poorer performance than when the monitor image was inverted around the *y*-axis to correct for the fulcrum effect. In addition, inverting the normal laparoscopic image around the *y*-axis accelerates the learning of novice subjects. Subsequently, the effect of such an inversion on the performance of experienced surgeons has also been shown by Crothers et al. [29]. They indicated that The *y*-axis-inverted image has a detrimental effect on the performance of experienced surgeons who have automated to the “fulcrum effect” of the abdominal wall on instrument manipulation. *y*-axis-image inversion was found to facilitate significant learning trends, regardless of the participants' level of experience.

Although surgical performance improves with repetitions as has been known, there is limited information about how much repetition is needed for maximal improvement of the expected improvement. For this reason, Voitk et al. [35] carried out a study under stable condition to estimate the approximate number of operations until ceases and the magnitude of that improvement. Based on their findings, improvement persists for about 200 operations, resulting in a 40% reduction in laparoscopic cholecystectomy operative time. The primary mechanism of improvement seems to be an ability to deal more effectively with difficult cases.

In laparoscopic surgery training field, Matern et al. [36] used learning curves as a criterion to compare two different types of handles via virtual reality simulation. According to the results, intraday learning curves were found over the entire test period of 13 days for clipping and cutting times measured. The initially unskilled volunteers quickly learned the task and were specialized for this surgical procedure after only a few days.

In summing up the results of the previous studies, the learning curve is a verified and efficient tool in evaluation laparoscopic operation or training performance. To novices or surgical trainees, adapting the scale of their movements to match the scale of the task on the display and the perceived inversion of movement from the handles to the working end of the instrument through repeated attempts to essential activities in laparoscopic procedures is their greatest preoccupation. The target acquisition task is one of the most essential activities in laparoscopy. Surgeons move instruments to the organ or tissue to be operated in three-dimensional movements. However, previous work has not examined a wide range of three-dimensional movement angles and has not considered the effects of visual magnification, which we see as the most common usage scenario. Therefore, the second purpose of this study was to investigate systemically the effects of three-dimensional movement angles on target acquisition task performance under the situation where depth perception is impaired and degrees of freedom for instruments are limited. Furthermore, the learning curve was used in this study to test the effects of essential movement characteristics on learning performance of target acquisition task. It was hypothesized that the performance were affected significantly by three-dimensional movement angles and there were significant difference between essential movement characteristics on learning performance. For this reason, the current study applied Fitts' law [37] to this investigation.

In a study of hand-held tools in visually controlled movements, Fitts [37] conducted an experimental task to quantify the accuracy and performance of the movement. In the Fitts' paradigm, the index of difficulty (ID) was defined by the distance to the target and the size of target. The relation between the ID and the MT was assumed to be linear. A typical example in the Fitts' experiment is a subject moving as rapidly as possible between two fixed targets of width (*W*) set a distance (*D*) apart and hitting the targets with a pointed stylus. The MT can be predicted by the following equation:

(1) $$\begin{array}{c} \text{MT}=a\;+b\;\log_{2}\left(\frac{2D}{W}\right), \end{array}$$

where *a* and *b* are empirical constants determined through linear regression. The log term is called the ID (in units of bits). The realization of movement in Fitts' model is analogous to the transmission of information with physical communication systems. In such systems, the amplitude of a transmitted signal is described as perturbed by noise that results in amplitude uncertainty. The effect is to limit the information capacity of a communications channel to some value less than its theoretical bandwidth. Shannon's Theorem 17 expresses the effective information capacity *C* (in bits/s) of a communications channel of band *W* (in s^−1^) as *C* = *W* log⁡_2_((*P* + *N*)/*N*), where *P* is the signal power and *N* is the noise power. 

Some variations of the law have been proposed by direct analogy with Shannon's Theorem 17 [38]. MacKenzie [39] developed an equation differing only in the formulations for ID as shown in (2):

(2) $$\begin{array}{c} \text{MT}=a+b{\;\log}_{2}\left(\frac{D}{W}+1.0\right). \end{array}$$

The benefit of this formulation is that it provides the best statistical fit, reflects the information theorem underlying Fitts' law, and always gives a positive ID. 

The relationship between speed and accuracy that has been documented in experimental psychology and human factors engineering was equally concerned by researchers and motor behaviorists. However, evaluating performance based only on movement time criteria is difficult when faced with disparities in errors rates. To reflect both speed and accuracy simultaneously, MacKenzie [40] developed a technique for adjusting effective width based on the distribution of “hits” for each condition to accommodate spatial variability or error in response. Adjusted width (effective width, *W*_*e*_) is used to define an effective index of difficulty.

In 1998, an ISO standard established to assist in evaluating a pointing device was ISO 9241, titled “Ergonomic design for office work with visual display terminals (VDTs)” [41]. Part 9, “Requirements for nonkeyboard input devices,” proposes throughput (TP) as a performance measurement derived from both speed and accuracy of response. A system with a higher TP means that the system has a higher ability to transmit information. The measure in “bits per second (bps)” is computed as Throughput = ID_*e*_/MT, where MT is the mean movement time for all trials within the same condition, and ID_*e*_ = log⁡_2_(*D*/*W*_*e*_ + 1). The ID_e_ is the effective index of difficulty calculated from distance and effective width (*W*_*e*_). *W*_*e*_ is the width of the distribution of participants selection coordinates over a sequence of trials computed from *W*_*e*_ = 4.133SD where SD is the standard deviation of the selection coordinates reflecting the over-shoot or undershoot of the individual movements about the mean in the direction of motion [42]. 

This measurement concept can be used not only in nonkeyboard input devices but also in performing the conventional Fitts' law linear regression with probe or stylus. Hence, this approach was adopted in the present study to evaluate performance.

The main merit of Fitts' law stems from its robustness as a quantitative description of target acquisition movements across a variety of movement contexts. Because of such robustness, the law is verified and serves as a useful tool for evaluating and comparing human and system performance in laparoscopy-related studies. For example, Lin et al. [43] conducted a Fitts' pointing task to investigate the effects of weight distribution of laparoscopic instruments on movement performance. Based on their findings, the middle position required the least time to manipulate the laparoscopic instrument in pointing tasks and also obtained the highest throughput and the total average throughput obtained in this study was 8.43 bps. However, this high throughput value was obtained under the condition in which participants used a laparoscopic instrument as an ordinary long hand-held tool to perform target acquisition tasks with direct vision. 

Herring and Hallbeck [44] applied Fitts' target acquisition tasks following the ISO 9241-9 procedure to test two electronic cursor control devices for use in an articulating powered laparoscopic tool. Subsequently, a similar study was developed to access the performance of four input devices which could replace the manual trackball in a powered laparoscopic tool [45]. The throughput for input devices reported in these two studies ranged from 0.91 to 1.39 bps. In these two studies, it should be noted that participants used input devices attached to the handle of fixed laparoscopic instruments to perform target acquisition tasks. 

In the industrial engineering field, engineers have always been concerned with predicting the time of human motion. Many successful prediction methodologies have been in use in industrial for decades. Because of the need for a publically available methodology for prediction of micro-miniature assembly times, a time prediction system was developed by Hancock et al. [46]. This system called MTM-M is unique in industrial engineering because it uses the Fitts' Index of Difficulty (ID) [37, 47] as a predictor of motion time. Subsequently, Langolf and Hancock [48] investigated the ultimate quality of the ID as a predictor of microscopic motion time and also investigated the effect of microscope power under conditions more carefully controlled than were possible in the MTM-M industrial studies. This laboratory study showed that proper transformations of other task variables can be used to quantitatively predict nearly all of the remaining motion time variance. However, these studies mainly focused on the characteristics of human motion patterns performed in conjunction with a binocular microscope; those done under a monocular device have not yet been much researched. 

Although, Fitts' law originally applies to one-dimensional movements, it has also widely been applied to two-dimensional pointing tasks on Human-Computer interactive systems. However, when performing discrete three-dimensional pointing movements, the control over the amplitude and the duration of the forces generated becomes more complicated with an increase of the dimensionality of the task or the number of degrees of freedom related to the participating muscles and joints. To perform a three-dimensional pointing task higher muscular force is required, leading to more variable movement trajectories and, hence, more variable pointing times [49]. Based on these insights, it was expected that the duration of three-dimensional pointing movements would be more variable and be affected more markedly by movement direction than the duration of one-dimensional and two-dimensional pointing tasks. For this reason, an attempt was made by Murata and Iwase [50] to examine this hypothesis and to extend the original Fitts' law to three-dimensional pointing tasks.

In Murata and Iwase [50] study, the conventional Fitts' model cannot adequately explain the variance in movement time in a real three-dimensional pointing task, as it does not take the direction of movement into account. The more variable movement trajectories and higher muscular forces caused by a more complicated system for controlling the *x*-, *y*-, and *z*- positions in a three-dimensional pointing task lead to more variable pointing times. The experimental data appear to validate the research hypothesis and highlight the necessity of constructing performance models that take the effects of movement direction into account.

Unfortunately, this investigation used index finger of subjects as the pointing tool, did not examine other hand-held instruments in a real three-dimensional pointing task. In particular, when a laparoscopic surgery was performed, surgeons used the laparoscopic instruments about 40 cm in length under a situation losing depth perception. Based on Murata and Iwase [50] findings, it would be expected that the duration of pointing movements with the fulcrum effect and indirect vision would be more variable and be affected markedly by movement direction than the duration of normal three-dimensional pointing tasks. 

The third purpose of the present study was to examine this hypothesis and, when it would be confirmed empirically, to extend the original Fitts' law to three-dimensional pointing tasks with the fulcrum effect and indirect vision. To anticipate, we realized this goal as follows. First, we used the conventional Fitts' model to predict movement time data collected in a three-dimensional pointing task under the fulcrum effect and indirect vision environment. The fit was suboptimal due to the variance present in the data and the dependency of movement time on movement direction. Based on these results, an extended laparoscopic three-dimensional model of Fitts' law was proposed, which was shown to describe the data markedly better than the conventional Fitts' model.

## 2. Methods

The present study aims to investigate the essential characteristics of a motor control model of instrument manipulation while performing laparoscopic surgery via Fitts' task [37, 47]. In order to achieve the objectives of this study, the Fitts' pointing task was conducted and described in detail in the following sections.

### 2.1. Ethics

The experiment was reviewed and approved by an Institutional Review Board. All individual participants in this study gave written informed consent prior to their participation and were free to withdraw from the study without prejudice.

### 2.2. Participants

Ten right-handed graduate students were recruited to participate in this experiment (nine males and one female). The mean age and standard deviation of participants are 25.7 years (range 22–31) and 2.8 years, respectively. All participants had normal or corrected-to-normal vision with no other physical impairments. None were from the medical school and thus all participants had no surgical experience.

### 2.3. Apparatus

**Figure 1:**
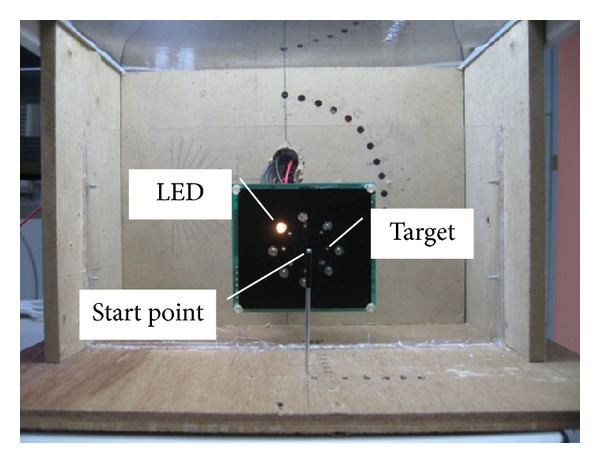
The laparoscopic simulated trainer (amplitude set at 60 mm).

**Figure 2:**
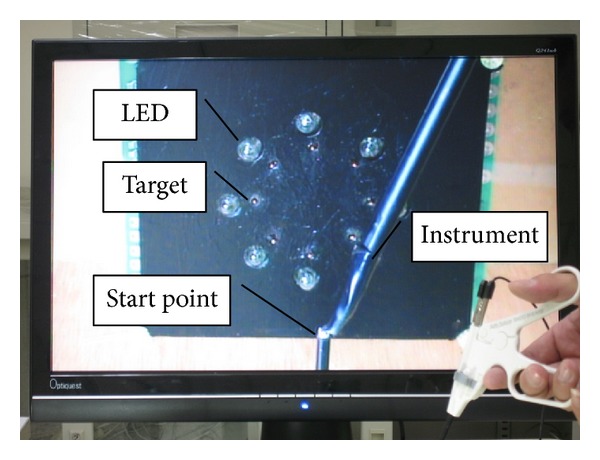
The image inside the trainer displayed by LCD screen.

**Figure 3:**
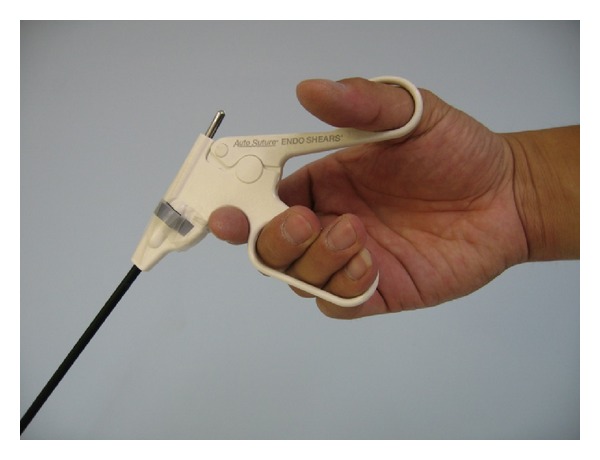
The laparoscopic instrument handle and its grip.

A laparoscopic simulated trainer developed for the present study was shown in Figure 1. This experimental configuration and type of movement were adapted from the Murata and Iwase [50] study. The board on which eight targets and eight LED (light-emitting diode) signal lights were located was placed vertically in the laparoscopic simulated trainer. As shown in Figure 2, eight metal cylinders, 1 mm high with a diameter of 1 mm, were used as the eight angular targets. The starting point was placed at the same height as the center of the board and moved away from the board according to the amplitude condition. As shown in Figure 3, the participant was instructed to place the jaw tip of the instrument at the starting point before the experimenter gave him the signal to start the movement. The effective amount of motion scaling is based on the location of the fulcrum for the instrument. For our study, the fulcrum was located approximately halfway up the instrument shaft. The main experimental task was that when an LED signal light was lighting up randomly, participants moved the instrument horizontally, vertically, and diagonally as soon as possible to the target next to the lightening LED signal light. Pointing movements were performed holding a laparoscopic instrument (ENDO SHEARS 5 mm 3/4′′ Curved Scissors, Autosuture) through a rubber diaphragm used as the patient' abdominal wall. The laparoscopic instrument, having a pistol configuration, was gripped with the third to fifth fingers (middle, ring, and little fingers) in an elliptic ring, with the thumb in the other, and the index finger against the top of the handle, creating a stable triangle for actuating the instrument in line with the forearm and hand (see Figure 3). A horizontal occluding board was used to prevent direct vision of the instrument displacement during performing tasks. However, participants could control the movement of the instrument visually through the video screen (Viewsonic Optiquest Q241wb 24′′ widescreen LCD monitor) located at 90 cm from the body. The LCD display surface was slanted 30° from the vertical plane toward the participant for easy observation. A digital camera (SONY, DCR-SR60) was set an angle of 45° (compared with horizontal plane) to record the displacement movement, and the latter was continuously and in real time visible on the screen. By changing the camera zoom, the amplitude of the movement perceived on the video screen was changed. The magnification had three levels, low, medium, and high; that is, the target diameter and movement amplitude were, respectively, 1.74, 3 or 4.26 times larger than the actual size. The whole experiment was controlled using a personal computer with a program developed in JAVA to set experimental parameters, give signals, and record data.

### 2.4. Experiment Design

Three within-subject factors were varied in the experiment. Independent variables were magnification (three levels: low, medium, and high), movement amplitude between starting point and target (five levels: 24.24, 33.11, 42.38, 51.92, and 61.61 mm), and angle to the target from the center of the board (*θ*) [(eight levels: 0° (right), 45° (upper right), 90° (upper), 135° (upper left), 180° (left), 225° (lower left), 270° (lower), and 315° (lower right)]. According to the amplitude condition, the distance between the pole and the board shown in Figure 1 was changed (20, 30, 40, 50, and 60 mm). Movement time (MT) and throughput (TP) that were recorded and computed after completing each task were used as dependent variables. Movement time was measured in milliseconds and throughput was calculated in accordance with the equation recommended by Soukoreff and MacKenzie [51]. The units of throughput are in bits per second (or bps).

In addition, the concept of the learning curve introduced by Wright [52] was employed. The learning curve described a basic theory for obtaining cost estimates based on repetitive production of airplane assemblies. It is recognized that repetition of the same operation results in less time or effort expended on that operation. Wright's original equation is *F*(*x*) = *a*_1_ *x* ^*b*^, where *F*(*x*) is the average cost of the first *x* units; *a*_1_ is the theoretical cost of the first production unit; *b* is a constant reflecting the rate costs decrease from unit to unit.

The equation transformed to fit the present study is *F*(*x*) = *A*_1_ *x* ^*B*^, where *F*(*x*) = average movement time of the first *x* trials, *A*_1_ is the average movement time of the first trial, and *B* is the learning curve coefficient reflecting the movement time decrease from trial to trial.

For calculating convenience, taking the logarithms of both sides of the equation reduces the equation mathematically to a straight line equation of the form log⁡(*F*(*x*)) = log⁡(*A*_1_) + *B*log⁡(*x*) or more commonly *Y* = *a* + *bX*. Least-squares linear regression is used to find the intercept (log⁡(*A*_1_)) and slop (*B*) parameters of the logarithmic learning curve. The conventional learning curve equation describes and models the observation that costs decrease by a constant percentage every time the quantity doubles. This constant percentage is called the learning rate (*r* = 2^*B*^) and the probability (1 − *r*) is defined as improvement rate (*p*) hence it can be considered as normalization and make it reasonable to compare across factors. For this reason, improvement rate (*p*) was adopted as dependent variables to examine the effects of factors on learning performance.

After computing with Microsoft Office Excel, the final data were transferred to the Minitab software for the statistical analysis. The movement time and throughput were tested using the Repeated Measure ANOVA procedure for significant statistical differences in means with significance level 0.05. Specific posthoc comparisons of independent variables were conducted by Tukey HSD test with a significance level 0.05. In the learning curve analyses, the Kruskal-Wallis procedure was used to test significant differences (*P* < 0.05) in medians of improvement rate (*p*). Finally, the linear regression was served as a test measuring the goodness of fit between movement time and the index of difficulty to develop the predict model. 

### 2.5. Procedure

Participants received practice trials before recorded movements to become familiar with the apparatus and experiment tasks. 

The order of 15 magnification by amplitude conditions was randomized. Within each magnification by amplitude combination, the order of pointing to the eight angles was randomized as well. Each participant performed a total of 1200 trials (3 magnifications × 5 amplitudes × 8 angles × 10 trials). 

Each condition was performed as follows: participants stood in front of the laparoscopic simulated trainer and held the laparoscopic instrument with their right hand. The jaw tip of instrument was placed at the starting position and the position of instrument handle was adjusted to be close to the subjects' elbow level to minimize discomfort and upper arm and shoulder muscle work [53]. At the beginning of each task, all of the eight signal LEDs were lit for 1 second to warn the participants that the task will start. Subsequently, the movements were performed from the starting point to the target next a lightening signal LED. The LED did not turn off until the specified target was pointed to correctly. There were ten pointing trials for each angular target. After the final specified target was pointed out correctly, all the eight LEDs turned on to indicate that this magnification-amplitude condition was complete. After completion of each condition, participants took a ten-minute break to avoid muscular fatigue. Participants were instructed to reach the targets by making a three-dimensional movement in the trainer as quickly and accurately as possible. The time when the tip of the instrument began to leave from the starting point was used as a criterion for movement onset, and when the tip of instrument reached the target, it was regarded as the end of pointing task. After completing each condition of task, the movement time was recorded for subsequent calculations and analyses.

## 3. Results

### 3.1. Adjustment of Data

A multiple comparisons test showed a significant decrease in movement time and a significant increase in throughput after the first trial (*P* < 0.05), but no significant difference over the last nine trials. Therefore, the first trial data was removed for all conditions from subsequent dependent variables analyses and Fitts' law model derivation. 

The data of fifteen conditions (magnification (4) × amplitude (5)) from the remaining nine trials were entered in a test for outliers, whereby trials with movement time more than three standard deviations from the mean were eliminated. Means and standard deviations were calculated for each combination of magnification, and amplitude. Of 10800 total trails, 177 (1.64%) qualified as outliers and were removed.

### 3.2. Dependent Variables Analysis

The Repeated Measure ANOVA procedure was performed to examine the effects of magnification, amplitude, and angle on the movement time and throughput. Specific post-hoc comparisons of independent variables were conducted by the Tukey HSD with a significance level 0.05.

#### 3.2.1. Movement Time

Mean movement time for the three magnifications, low, medium, and high, were 2476.0, 2266.1, and 2405.0 ms, respectively. There were significant differences between the magnifications in movement time (*F*_2,10494_ = 51.35,  *P* < 0.01). The movement time for the medium magnification was the fastest and the low magnification took the longest time. Further a Tukey HSD test dividing independent variable levels into groups is shown in Table 1 in which the means were not significantly different within the same group. The Tukey HSD test showed that the movement time of the three magnifications were significantly different from each other. 

There was a main effect of movement amplitude (*F*_4,10494_ = 35.50, *P* < 0.01) on the movement time. The movement time increased with the level of movement amplitude and the Tukey HSD test (see Table 1) indicated that the movement times significantly differed from each other except the 33.11 and 51.92 mm. 

There was a significant main effect of angle to the target (*F*_7,10494_ = 27.81, *P* < 0.01). Based on the Tukey HSD test results (see Table 1), movement times for the left (180°) and lower right (315°) were significantly longer than those for the six other conditions. Furthermore, the following comparisons were significant: movement time was longer for the 135° than for the 225, 0, 90, and 45; movement time was longer for the 270° and 225° than for the 45°. 

Significant interactions were found between magnification and amplitude (*F*_8, 10494_ = 4.54, *P* < 0.01) and between angle and amplitude distance (*F*_28, 10494_ = 2.39, *P* < 0.01) in movement time. It showed that the movement time of all three magnifications increased with the level of amplitude. Furthermore, multiple comparisons results showed that the movement of medium magnification was significant smaller than two other magnification conditions in four movement amplitudes, 24.41, 33.11, 51.92, and 61.61 mm. It was observed that the movement time of all eight magnifications increased with the level of amplitude.

#### 3.2.2. Throughput

Throughput computed for the medium magnification at 2.7436 bps was the highest as presented in Table 2. For the low and high magnifications, they were 2.5496 and 2.5456 bps, respectively. The ANOVA test result showed that the main effect of magnification was significant (*F*_2,10494_ = 37.23, *P* < 0.01). Tukey HSD test results were shown in Table 2, which indicated that the throughput for the medium magnification was higher than the low and high magnification statistically, but there was no significant difference between the low and high magnification. 

There was also a significant difference throughput between movement amplitude (*F*_4,10494_ = 32.50, *P* < 0.01). The throughput increased with the level of movement amplitude and the Tukey HSD test result indicated that the movement time of the movement amplitude 51.92 mm was the highest and there were significant differences between three remaining movement amplitude groups (see Table 2).

As shown in Table 2, there was a significant main effect of angle to the target (*F*_7,10494_ = 26.89, *P* < 0.01). Based on the Tukey HSD test results showed in Table 2, it was found that the throughput for the upper right (45°) was the highest and for the left (180°) was the lowest. Furthermore, the remaining six angle to the target conditions were divided into three groups, *B* (315°), *C* (135°), and *D* (0°, 90°, 225°, and 270°).

In addition, there were significant magnification by movement amplitude (*F*_8, 10494_ = 5.57, *P* < 0.01) and angle to target by movement amplitude (*F*_8, 10494_ = 1.94, *P* < 0.01) interactions in throughput. It showed that the throughput of magnifications and angles increased with the level of amplitude.

### 3.3. Learning Performance Analysis

#### 3.3.1. Overall Learning Performance

In this experiment, participants performed the task for 120 conditions (magnification (4) × amplitude (5) × angle (8)) of ten trials each. Based on other similar experiments, it was hypothesized that participants would have achieved a criterion level of practice by the final trial. In other words, no significant improvement in movement time and throughput would be shown in the tenth trial.

**Figure 4:**
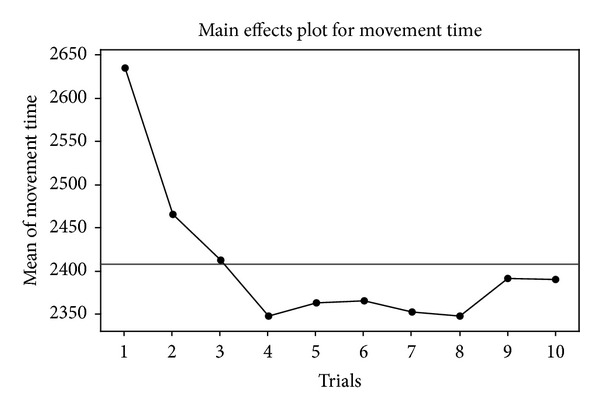
Main effect plot of trials for movement time.

An ANOVA was performed to examine the overall learning effect on movement time and throughput. Mean movement time tended to decrease as a function of trials is plotted in Figure 4. Mean movement times for the ten trials were 2636.1, 2465.8, 2413.4, 2347.9, 2364.1, 2366.1, 2353.5, 2348.8, 2391.7, and 2391.1 ms, respectively. There were significant differences between the trials in movement time (*F*_9, 11769_ = 10.94, *P* < 0.001). The post-hoc Tukey HSD test showed that the movement time of the first trial was significantly longer than the other nine trials, but there were no significant differences between the second to the tenth trials (Table 3). This result implied that the learning effect mitigated after the second trail and the movement maintained stable. 

**Figure 5:**
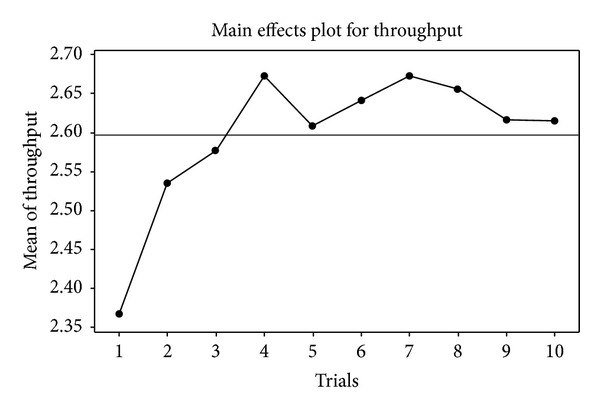
Main effect plot of trials for throughput.

Throughput computed for the first trial at 2.3676 bps was the lowest as presented in Figure 5. For the second to the tenth trials, they were 2.5357, 2.5776, 2.6735, 2.6083, 2.6420, 2.6738, 2.6561, 2.6166, and 2.6150 bps, respectively. Mean throughput tended to increase as a function of trials. A main effect of trials was found significantly (*F*_9,11769_ = 8.96, *P* < 0.001). Tukey HSD test results were shown in Table 3, which indicated that the throughput for the first trial was lower than nine other trails, and the second trial was lower than the seventh trial. Synthesizing Figure 5 and Table 3, the throughput was influenced by trials, but this effect tended to decrease after the third trial. 

#### 3.3.2. Effects of the Essential Movement Characteristics Learning Performance

To further investigate the effects of essential movement characteristics, magnification, amplitude, and angle, on learning performance of movement time and throughput, improvement rates (*p*) were used as dependent variables to perform subsequent analyses.

Except the angle 180° and 315°, with increases trials, movement time decreases and throughput increased in all other conditions. For this reason, it is useful to transform movement time and throughput into learning curve model and then using the Kruskal-Wallis procedure to test significant differences (*P* < 0.05) in medians of improvement rate (*p*). It would help us to figure out the effects of the factors on learning performance.

The means and standard deviations of the improvement rate for movement time and throughput are shown in Table 4. Mean improvement rate of movement time for the three factors, amplitude, angle, and magnification, were 2.705%, 2.689%, and 2.761%, respectively. There were no significant differences between these three factors in improvement rate of movement time. Mean improvement rate of throughput was the highest for the angle (2.977%), followed by magnification (2.874%), and then the lowest amplitude (2.872%). As shown in Table 4, the improvement rate of throughput did not reach significant main effect result for the factors. 

However, it is noteworthy that both movement time and throughput logarithmic transformed learning curve of the angle 180° and 315° did not pass the linear regression test. In other words, under these two angle-to-target conditions, participants did not improve both in movement time and throughput after performing ten trials pointing task.

### 3.4. Predicting Model Construction

#### 3.4.1. Fitting the Data to the Conventional Fitts' Model

First, the data were modeled using the following conventional Fitts' model (2) [39]:

(3) $$\begin{array}{c} \text{MT}=a+b\;\log_{2}\left(\frac{D}{W}+1\right), \end{array}$$

where MT represents the time to move the laparoscopic instrument from the starting point to the target and *D* and *W* are the distance from the starting point to the target and the size (diameter) of the target, respectively. The term log⁡_2_(*D*/*W* + 1) is the index of difficulty carrying the unit of bits. The four movement amplitude and a constant target diameter produced four IDs ranged from 4.67 to 5.97 bits. Finally, the parameters *a* and *b* are empirical constants to be determined through linear regression.

**Figure 6:**
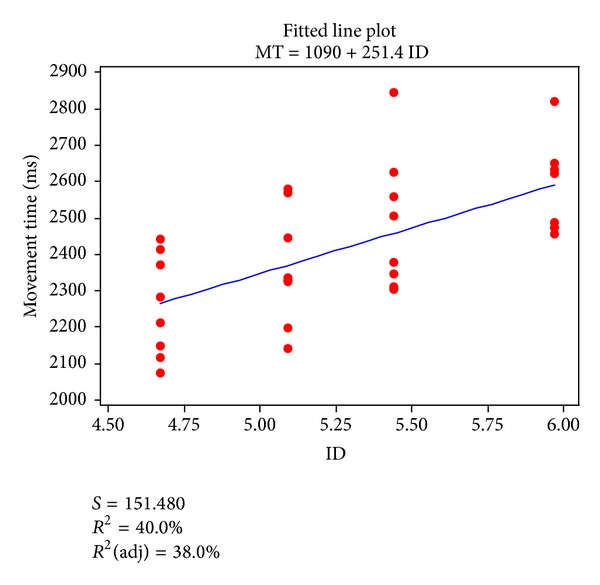
Relationship between index of difficult (ID) and mean movement time (averaged over participants).

As described in data adjustment, the first trial was excluded from the analysis. For the nine remaining trials, the mean movement time was calculated for each index of difficulty, pooled over all angle conditions and participants. The *r* ^2^ of the linear regression between mean movement time and index of difficulty was 0.40 (see Figure 6). The 95% confidence intervals of the slope and intercept for the regression line were [136.66, 366.06] and [480.62, 1699.68], respectively, while the standard error of the difference between the measured movement time and the value predicted by linear regression was 151.48 ms. On the basis of this relatively poor fit it can be concluded that there is still substantial room for improving upon the conventional Fitts' model when it comes to the description of laparoscopic display-controlled movements.

#### 3.4.2. Extending Fitts' Law to a Laparoscopic Display-Controlled Task

As described in Section 3.2, a main effect of angle to target was observed and these results indicated the presence of a systematic relationship between movement time and angle to the target. These implied that a model taking angle into account will lead to a better performance model than the conventional Fitts' model. Although angle to target has some effects on the movement time in two-dimensional tasks [54–56], there are few explicit models that take these effects into account. However, angle to target seems to be an important factor in performance modeling, especially the modeling of three-dimensional movement tasks. For this reason, Murata and Iwase [50] proposed an extended Fitts' model where taken into account by incorporating *θ* for three-dimensional movement. In order to further find a meaningful extension of the conventional Fitts' model to laparoscopic display-controlled movements, the formula proposed by Murata and Iwase [50] was adopted. Based on the data form the present study, the ID was revised using the following formula:

(4) $$\begin{array}{c} \text{I}{\text{D}}_{L}=\log_{2}\left(\frac{A}{W}+1\right)-c\cos\;\theta , \end{array}$$

where *c* is an arbitrary constant to be determined through linear regression. 

**Figure 7:**
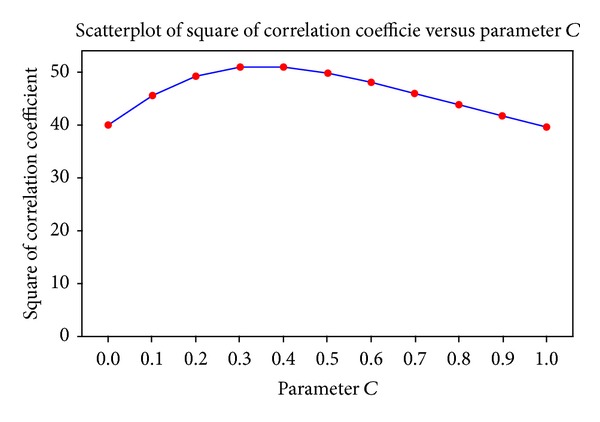
Squared correlation coefficients as a function parameter *c* for fitting the data in Figure 6 to the extended Fitts' model.

**Figure 8:**
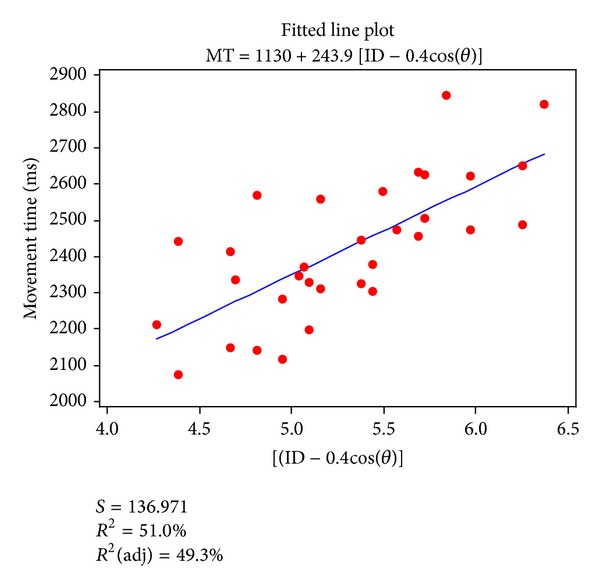
Mean movement time (averaged over participants) as a function of the index of difficulty (ID_*L*_) in the extended Fitts' model (*r* ^2^ = 0.51, *c* = 0.4).

For several values of *c*, the relationship between ID_*L*_ and movement time was established by means of linear regression.  Figure 7 shows that the highest *r* ^2^ (0.51) was found for *c* = 0.4. The fit to the experimental data was improved by using the index of difficulty ID_*L*_, which incorporates the effect of angle on movement time, and by using the value of *c* producing the highest *r* ^2^ (Figure 8). The optimal values of *c* differed for the experimental data of the individual subjects.

The fit to the obtained movement times was better when extended laparoscopic display-controlled modeling was applied. The 95% confidence intervals of the slope and the intercept for the regression line for the laparoscopic display-controlled model were [154.74, 333.16] and [654.92, 1604.22], respectively. These confidence intervals are narrower than for the conventional Fitts' model. The standard error of the difference between the measured movement time and the value predicted by the fit to the laparoscopic display-controlled modeling was 136.97 ms, which is smaller than that (151.48 ms) of the fits to conventional Fitts' model. Collectively, these results clearly indicate that the extended model of Fitts' law better predicts the duration of laparoscopic display-controlled movements than the conventional Fitts' model.

## 4. Discussions

### 4.1. Dependent Variables Analysis

#### 4.1.1. Movement Time

The analytical results of this study indicated that as the movement amplitude increased, movement time significantly increased, and vice versa. This outcome is in agreement with previous studies of Fitts' law published in recent decades [30, 43, 48, 57]. This finding also demonstrates that the movement time rises with the level of movement amplitude when a video-controlled pointing task with a long-shift laparoscopic instrument is performed.

However, it is noteworthy that the mean movement time of 2407.7 ms obtained in the present study is much higher than that observed when the movement was carried out either in the normal condition or in microscopic work. Indeed, former experiments conducted in the directly visually controlled condition showed that the mean movement time was 345.2 ms for a laparoscopic instrument pointing [43] and was 476.9 ms for a long dowel pointing [57]. The movement amplitude of the former ranged from 140 mm to 370 mm, and the latter values were refined from the conditions where the probe length = 40 mm and movement amplitude ranged from 100 to 400 mm. It is obvious that the movement amplitude of the present study was shorter but used a much longer movement time than that of these two studies.

In microscope working conditions, the mean movement time was 183.2 ms for Langolf et al. [30] (where amplitude ranged from 2.5 to 12.7 mm) and was 676.1 ms for Langolf and Hancock [48] (where amplitude ranged from 2.54 to 7.62 mm). Under similar ranged movement amplitude, the mean movement time was approximately between 3.5 and 12 times longer than that of these two studies. 

One possible reason for the discrepancy is the lack of accurate depth perception. When performing a three-dimensional pointing task through a two-dimensional image, in order to overcome this lack of depth perception, participants had to find the position of the instrument by touching the board that targets attached to and then moving the instrument jaw to touch targets along the board. As a result, pointing tasks that take seconds during direct visual feedback can take minutes during display control [34]. 

Another reason for the discrepancy might be due to the awkwardness of the surgical instruments themselves [58]. In particular, because the instruments are introduced through “keyholes,” participants must deal with three important limitations: (a) their instruments must be long (up to ~50 cm); (b) rigid instruments lose two translational degrees of freedom (*x*- and *y*-axes in the plane of the rubber diaphragm surface) due to constraints associated with the entry portal; and (c) the transverse translational motions of the tip of their instruments and their hands must be reversed, again because of the keyhole constraint. In combination, the loss of feel, the added motion constraints, and the cognitive remapping dramatically increase the difficulty of surgical manipulations.

There were significant differences between the magnification levels in movement time, based on the analytical results. Movement time for the medium magnification was the fastest, and the low magnification took the longest time. These results are inconsistent with those of previous research. In the studies of Langolf and Hancock [48] and Ferrel et al. [59], the results showed that magnification does not significantly affect movement time. However, the movement time significantly decreased as magnification levels increased in Ellis et al. [60]. One possible reason for result discrepancies between the present and the three previous studies is due to the apparatus and tasks. 

Unlike the studies where participants moved a part with a tweezer [48], performed tasks by holding a stylus along the horizontal plane [59], or carried out a Fitts' task on a PDA by manipulating a tele-robot [60], the task of this study was to maneuver a long-shift laparoscopic instrument in a three-dimensional environment via two-dimensional video images. In contrast with those studies, it was more complicated and difficult for participants to adjust eye-hand coordination over this range without affecting their performance. For this reason, the magnification levels became a critical factor affecting the performance of pointing tasks. It was useful for participants to have a clear look at the targets and instrument jaw and to distinguish the relative position from them by magnifying the picture inside the laparoscopic simulated trainer. However, the movement time did not decrease with the level of magnification. 

The movement time for three magnification levels was revealed as a V-shape. A possible reason for resulting in this shape is the control-display gain setting. The gain settings involve a trade-off between gross-positioning time (getting to the vicinity of a target) and fine-positioning time (the final acquisition), an effect first noted by Jenkins and Connor [61]. With a high-gain (low magnification) setting, participants can quickly maneuver the instrument jaw to the vicinity of the target (gross-positioning), but final acquisition of the target is exacerbated by the difficulty in precisely controlling the final position of the instrument jaw (fine positioning). In other words, in low magnification setting, moving phase may be fast but positioning phase would be slow. Low-gain settings (high magnification), on the other hand, facilitate fine positioning of the instrument jaw, but increase the time to advance the instrument jaw over large distances (gross-positioning) [62]. In other words, in high magnification setting, there was a slow movement phase with a fast positioning phase. Total movement was the summation of these two phases. Accot and Zhai [63] and Jellinek and Card [64] found that very low and very high CD gains reduced performance creating a U-shaped profile for movement time versus CD gain. In the present study, the medium level obtaining the lowest movement time seems to be a better magnification setting.

The results of this experiment showed that the angle of approach significantly affects the time required to manipulate a laparoscopic instrument in a three-dimensional environment by monitoring with two-dimension images. Further analytic results showed that movement times for movements along the four diagonals (45°, 135°, 225°, and 315°) were not significantly different from those for the two horizontal (0° and 180°) and two vertical (90° and 270°) directions. However, the following comparisons were significant: movement time was shorter for the right conditions (0°, 45°, and 315°) than for the left conditions (135°, 180°, and 225°); movement time was shorter for the upper conditions (45°, 90°, and 135°) than for the lower conditions (225°, 270°, and 315°). These tendencies may be attributed to the relative distance between targets and the entry portal. Because the entry portal was located at the right side for the right-handed participants, this setting consequently increased the distance to the lower and left targets. For this reason, it was observed that participants used more elbow extension for lower and left conditions than for upper and right conditions to make the laparoscopic instrument into the trainer. These results can be interpreted according to the results obtained in the study by Langolf et al. [30], in which movement time and throughput for both the forearm and the wrist was lower than those for only the wrist. In addition, the movement time for eight angle conditions in the present study can be described by a negative cosine wave.

However, these results were inconsistent with the two-dimensional computer-based cursor movements [54] and the three-dimensional finger pointing tasks [50]. In Whisenand and Emurian's [54] study, movements were generally faster along the two vertical and two horizontal directions, in comparison with movements along the four diagonal directions. In the Murata and Iwase [50] study, movement times to targets in the upper directions (upper, upper left, and right) were tended to be longer than movement times to targets in the lower directions (lower, lower left, and lower right), and the movement time for each subject and the mean value of all subjects had forms that could be described by a sinusoidal wave. 

A possible explanation for the inconsistency between these two studies and the present one is the factor of dimensionality. In contrast to the two-dimensional computer-based cursor pointing tasks, the control over the amplitude and the duration of the forces generated becomes more complicated with an increase in the task's dimensionality or in the number of degrees of freedom related to the participating muscles and joints. To perform a three-dimensional pointing task, higher muscular force is required, leading to more variable movement trajectories and, hence, more variable pointing times [49]. 

In comparison to the three-dimensional finger pointing tasks [50], with a decrease in dimensionality of the depth cue due to image transmission and with the entry portal limits of the two translational degrees of freedom (*x*- and *y*-axes in the plane of the rubber diaphragm surface), the task in this study becomes more difficult and complicated.

#### 4.1.2. Throughput

Based on the experimental results, the throughput had a tendency to increase with the level of amplitude. This implies that when the amplitude increases, participants have enough ability to process information produced by index of difficulty. 

The average throughput (2.62 bps) obtained in this study is much lower than that observed in Lin et al. [43], and in Baird et al [57]. Former experiments conducted in the directly visually controlled condition showed that mean throughput was 8.43 bps for a laparoscopic instrument pointing [43] and was 8.06 bps for a long dowel pointing [57]. In comparison with the study where hand-pointing movement was visually controlled through a video display [59], the throughput estimated from the tenth trial for a block and a random trial presentation were 7.26 bps and 7.56 bps, respectively. These values are almost three times more than those obtained in the present study. A brief summarization of these comparison results is that the information processing capacity of a video-controlled laparoscopic instrument pointing task is one-third of that obtained from a directly visually controlled task with a long instrument or a video-controlled hand-pointing task. As described previously, a possible reason for the discrepancy is the lack of accurate depth perception and limitations induced by instrument manipulation. 

In a microscope work condition, the throughput for the fingers, the wrist, and the arm were 38, 23, and 10 bps, respectively [30]. However, these results were skeptical, according to Balakrishnan and MacKenzie [65]. The processing capacity for the fingers and wrist were among the highest ever reported with Fitts' law. Additionally, they were obtained in data from only three participants and from regression models based on only three points each. For this reason, these results were interpreted with caution and considered useful merely as reference information rather than for purposes of comparison.

Generally speaking, the throughput under a given index of difficulty has a tendency contrary to movement time, meaning that higher movement time results in lower throughput, and vice versa. In magnification levels, the medium magnification seems to be an optimal tradeoff between the gross movement and fine movement, as the throughput for medium magnification was highest among the three levels. However, the throughput for the low and high magnification, considered as the two end points of the U-shaped influence, are not significantly different. 

The results of this experiment showed that the angle of approach significantly affects the throughput of manipulating a laparoscopic instrument in a three-dimensional environment by monitoring two-dimensional images. Further analytic results showed that throughputs for movements along the four diagonals were not significantly different from those for the two horizontal and two vertical directions. However, the following comparisons were significant: throughput was higher for the right condition than for the left condition; throughput was higher for the upper condition than for the lower condition. As mentioned previously, one possible reason was the relative distance between targets and the entry portal. Because the entry portal was located at the right side for the right-handed participants, this setting consequently increased the distance to the lower and left targets. For this reason, participants had to use more elbow extension for lower and left conditions than for upper and right conditions to make the laparoscopic instrument into the trainer. These results can be interpreted by the Langolf et al. [30] study where throughput for both the forearm and the wrist was lower than those for only the wrist.

### 4.2. Learning Performance Analysis

#### 4.2.1. Overall Learning Performance

Based on the analytic results, movement time was 2632.1 ms for the first trial. After the first trial, movement time plateaued to a 2382.5 ms average, which remained steady for the next nine trials, comprising a reduction of 10.5% after the first trial. The decrease in movement time was significant (*P* < 0.05) between the first and second trials. In addition, after the first trial in throughput, a significant cut-off (*P* < 0.05) again seems to occur. The throughput for the first trial was 2.6376 bps, which plateaued to a 2.662 bps average, remaining steady for the next nine trials, comprising an improvement of 12.4% after the first trial. These findings are in accord with the results of previous Ferrel et al. [59] study, despite the fact is that this study used a very different pointing task method. This situation implies that whether a laparoscopic instrument or hand was used for a video-controlled pointing task, participants seemed adaptive to this control setting in regard to movement time and throughput after the first trial. 

The overall improvement rates for movement time and throughput were 2.757% and 2.864%, respectively. These results imply that movement time and throughput will improve by a constant percentage every time the trial doubles. 

#### 4.2.2. Effect of the Factors on Learning Performance

According to the results of a Kruskal-Wallis test in medians of improvement rate (*p*) for movement time and throughput, there were no significant differences between magnification, amplitude, and angle. However, it is noteworthy that, in the conditions of the angles 180° and 315° participants did not improve either in movement time or throughput with an increase of trial. The standard deviation of the angle condition shown in Table 4 is relatively higher than that of amplitude and magnification. Although the main effects between these three factors were not significant, it seems that participants did not adapt quickly to the change of direction when performing the video-controlled movement tasks with a laparoscopic instrument. These findings suggest that, when designing a laparoscopic training program, it can be expected that trainees' angular movement ability will be strengthened with more practice. Furthermore, the findings also suggest that surgeons should carefully consider the location of the entry portal, to avoid involving the angle 315° in the surgery. Participants approaching the angle of 315°, which not only took higher movement time but also obtained lower throughput than from other angle conditions, were likely to increase surgical risk for patients.

### 4.3. Predicting Model Construction

Typically, in two-dimensional pointing tasks on computer-based systems, the correlation coefficient *r* between the index of difficulty and pointing time is much larger than that shown in Figure 6 [39, 40, 55, 56, 66]. Jagacinski and Monk [55] and Boritz et al. [56] investigated how directional mouse movement affects pointing time and showed that the pointing times differed significantly across conditions of directional movement. In these studies, however, the performance modeling was conducted by pooling all directional mouse movements. Nevertheless, the contribution *r* ^2^ was much higher than in the present study (0.990 versus 0.51). This fact suggests that the effect of directional movement is not very prominent in two-dimensional computer-based pointing tasks. Hence, it appears reasonable to model the pointing time in such tasks with the conventional Fitts' model.

In the present study, these findings were in line with the previous three-dimensional hand pointing experiment [50], in which movement time was significantly affected by movement direction. Movement times to targets in the lower directions (225°, 270°, and 315°) tended to be longer than movement times to targets in the upper directions (45°, 90 °, and 135°). Additionally, movement times to targets in the left directions (135°, 180°, and 225°) tended to be longer than movement times to targets in the right directions (0°, 45°, and 315°). Thus, contrary to two-dimensional computer-based pointing tasks, the conventional Fitts' model cannot adequately explain the variance in movement time in a real-world three-dimensional pointing task, as it does not take into account the direction of movement [50].

In contrast to the Murata and Iwase [50] study, the results of the present study show that the control over amplitude and duration of the forces applied during a video-controlled movement becomes more complicated because of the lack of depth perception and the degrees of freedom restricted by the entry portal. When performing discrete aiming movements, limb displacements are achieved by generating adequately scaled and timed activity in both agonist and antagonist muscles [49, 67]. The more variable movement trajectories and higher muscular forces required by a more complicated system for controlling the *x*-, *y*-, and *z*-positions in a video-controlled pointing task with a laparoscopic instrument lead to more variable pointing times [49]. This dynamic can be observed from the fact that the contribution *r* ^2^ (0.51) of the present study was lower than that (0.726) of the Murata and Iwase [50] study, even though the direction was taken into account. The experimental data appear to support Murata and Iwase's [50] study and highlight the necessity of constructing performance models that take into account the effects of movement direction. 

The mean movement time of all subjects can be described by a negative cosine wave. These characteristics were taken into account in reconstructing the index of difficulty according to (4). Specifically, a directional term (−cos⁡*θ*) was added to the conventional index of difficulty ID to reduce the variance in the modeling. On the basis of analyses of the contribution of *r* ^2^, of standard error, and of the confidence interval of the data fitting, it was demonstrated that the predictive power of the extended performance model was superior to that of the conventional Fitts' model (compare Figures 8 and 6).

This predictive model can analyze operator movement during surgical procedures to estimate movement time under particular tissue dimensions, instrument movement amplitude, and direction. Moreover, movement time can be normalized to establish training standards. Finally, the method can improve resource allocation by avoiding extremely time-consuming procedures and can enhance quality and performance while training.

Because of the importance of controlling potentially confounding variables, we used well-designed laboratory experiments to investigate the essential characteristics of laparoscopic instrument movement control and its learning effect and to develop a model for predicting instrument movement time. Our findings are essential contributions to a real laparoscopic field. However, due to experimental limitations, using laparoscopic technology in this study could only test visible variables or extending ones such as movement time and throughput without any consideration about the real tactile sense. For example, there exist soft or elastic organs and tissues in human body. In order to sufficiently understand the movement-control characteristics of the laparoscopic surgery, the consideration about the tactile sense are suggested for further research. In addition, although the present study demonstrated some positive results to support the purposes and hypotheses, the major limitation of this study is that all results were based on 10 graduate students without surgical background. Based on a study by Chien et al. [68], surgical experience did play an important role when examining accuracy and speed trade-off. For this reason, more levels of participants' surgical experience should be taken into consideration in future research. 

## 5. Conclusion

The results lead to several conclusions regarding effects of essential characteristics of laparoscopic instrument movement control on motor performance and learning performance. First, the magnification levels indeed affected the movement time and the throughput when performing video-controlled pointing tasks with a long hand-held laparoscopic instrument. Second, the movement time increased with an increase of movement amplitude during the performance of video-controlled pointing tasks. The mean movement time obtained in the present study was 5 to 7 times longer than that in direct visual studies and was approximately 3.5 to 12 times longer than those in stereoscopic microscope work conditions. Third, the movement time was discovered to be shorter for the upper and right conditions than for the lower and left conditions. The control performance was the worst when moving to angles of 180° and 315°. These two angular directions may result in surgical risk to patients. Fourth, the movement time and the throughput improved with the level of trial. It appeared that participants were able to adapt quickly to this video-controlled task environment. The overall improvement rates for movement time and throughput were 2.757% and 2.864%, respectively. Furthermore, it seems that participants did not adapt quickly to the change of movement direction. Therefore, it was suggested, when designing a laparoscopic training program, to strengthen the angular movement ability of trainees with more practice.

Finally, in the present study, movement time was affected significantly by the directions of movement. The conventional Fitts' model cannot adequately explain the variance in movement time in a video-controlled pointing task. After taking directional characteristics into account when recasting the index of difficulty, the predictive model proposed in this study accounted for 51 percent of the variance in movement time.

## Figures and Tables

**Table 1:** Means, ANOVA, and Tukey HSD test results of movement time.

	Movement time (ms)			
	Level	Mean^a^	*F*_*n*,*m*_	*P* value
Magnification	Low	2476.0^C^	*F*_2,10494_ 51.35	<0.001
	Medium	2266.1^A^		
	High	2405.0^B^		

Amplitude	24.41	2245.8^A^	*F*_4,10494_ 35.50	<0.001
	33.11	2342.8^B^		
	42.38	2452.3^C^		
	51.92	2329.5^B^		
	61.61	2540.6^D^		

Angle	0	2302.3^A, B^	*F*_7,10494_ 27.81	<0.001
	45	2240.8^A^		
	90	2285.1^A, B^		
	135	2416.0^C^		
	180	2603.3^D^		
	225	2323.0^B^		
	270	2353.3^B, C^		
	315	2543.3^D^		

^a^A, B, C, or D is group divided by Tukey HSD tests. The means in the same group are not significantly different by the test.

**Table 2:** Means, ANOVA, and Tukey HSD test results of throughput.

	Throughput (bits/s)			
	Level	Mean^a^	*F*_*n*,*m*_	*P* value
Magnification	Low	2.5469^A^	*F*_2,10494_ 37.23	<0.001
	Medium	2.7436^B^		
	High	2.5756^A^		

Amplitude	24.41	2.4568^A^	*F*_4,10494_ 32.50	<0.001
	33.11	2.5726^B^		
	42.38	2.5920^B^		
	51.92	2.7995^D^		
	61.61	2.6889^C^		

Angle	0	2.6912^D^	*F*_7,10494_ 26.89	<0.001
	45	2.8152^E^		
	90	2.7168^D^		
	135	2.5782^C^		
	180	2.3528^A^		
	225	2.6622^D^		
	270	2.6796^D^		
	315	2.4714^B^		

^a^A, B, C, D, or E is group divided by Tukey HSD tests. The means in the same group are not significantly different by the test.

**Table 3:** Means, ANOVA, and Tukey HSD test results of movement time and throughput.

	Movement time (ms)				Throughput (bits/s)			
	Level	Mean^a^	*F*_*n*,*m*_	*P* value	Level	Mean^a^	*F*_*n*,*m*_	*P* value
Trials	1	2636.1^A^	*F*_9,11769_ 10.94	0.000	1	2.3676^A^	*F*_9,11769_ 8.96	0.000
	2	2465.8^B^			2	2.5357^B^		
	3	2413.4^B^			3	2.5776^B,C^		
	4	2347.9^B^			4	2.6735^B,C^		
	5	2364.1^B^			5	2.6083^B,C^		
	6	2366.1^B^			6	2.6420^B,C^		
	7	2353.5^B^			7	2.6738^C^		
	8	2348.8^B^			8	2.6561^B,C^		
	9	2391.7^B^			9	2.6166^B,C^		
	10	2391.1^B^			10	2.6150^B,C^		

^a^A, B, or C is group divided by Tukey HSD tests. The means in the same group are not significantly different by the test.

**Table 4:** Means, standard deviations, and Kruskal-Wallis test results of improvement rate of movement time and throughput.

	Improvement rate of movement time (%)					Improvement rate of throughput (%)				
	Level	Mean	StDev	*H*	*P* value	Level	Mean	StDev	*H*	*P* value
Factor	Amplitude	2.705	1.119	0.04	0.979	Amplitude	2.872	1.415	0.04	0.982
	Angle	2.689	2.067			Angle	2.977	2.412		
	Magnification	2.761	0.387			Magnification	2.874	0.557		
